# Strangulated Femoral Hernia

**Published:** 1874-09-01

**Authors:** Charles Winne

**Affiliations:** Sandwich, Illinois


### STRANGULATED FEMORAL HERNIA.

REPORT OF OPERATION BY CHARLES WINNE, M. D., SANDWICH, ILLINOIS.

PATIENT, Mrs. C., forty-three years of age, native of the United States; mother of four children; of spare figure and of average height; general health good; bowels usually costive. Has had right femoral hernia for twelve years, descending and returning spontaneously, or readily reduced by assuming the recumbent position and slight manipulation; has worn a truss for the last four years.

Sept. 13th, 1873.—Saturday—Patient arose very early in the morning and was working in the milk-room, when she felt a severe pain in the region of her hernia. She immediately attempted to return it in order that she might replace her truss, which had been forgotten upon arising, but failed after repeated attempts in bed. The pain increasing, with great nausea, she sent for her family attendant, a homœopathist, at eight A. M., who came and reduced the hernia, as he said, the patient all the time protesting that he had not returned it. He then gave her a large dose of castor oil and left, telling her it would be "all right." He was called three times during the day, and each time, after repeated attempts and failures at reduction, he ordered large doses of castor oil and opium, and left: his patient suffering the most excruciating pain and vomiting almost constantly.

Sept. 14th.—Sunday, one A. M.—Dr. Winne was called to see Mrs. C., the homœopathist having been discharged at twelve (midnight). Dr. V. Vermilye was called in council. Patient had been suffering for twenty-one hours with a tumor as large as a hen's egg in the right femoral region; found her much prostrated; pulse frequent and irritable; respiration of a sighing character; patient in great agony; constant vomiting; face hard and pinched. Chloroform was given, the patient placed in proper position, and gentle attempts at reduction made. Failing in this, ice was applied, and farther attempts at reduction made. Still failing, at six A. M. an operation was decided upon. Two of Dr. Winne's office-students having arrived, the patient was fully anæsthetized, and an incision made transversely across the upper portion of the tumor, intersecting this by another carried down toward its base. The various layers were then cautiously divided down to the sac with the assistance of a grooved director, and attempts made to relieve the stricture without opening the sac, but without success. The sac was then carefully opened up to the point of stricture, and was found to contain a mass of omentum as large as a pullet's egg, underlying which was an intensely congested knuckle of intestine. The stricture was carefully divided with the knife, and the intestine and omentum returned within the cavity of the abdomen, and wound closed. After the operation the patient was very much exhausted and depressed. Her oft-repeated "Oh, I feel *so* bad," was relieved by a hypodermic injection of one-sixth of a grain of sulphate of morphia, and stimulants. Vomiting and nausea presisted for some time. Cold water dressings were applied to the wound for several days. The patient was kept quiet by hypodermics of morphia; bladder relieved by use of the catheter. Iced drinks were given the patient with lime-water, milk, raw eggs, etc. The patient rallied and did well until the fourth day, when she had symptoms of an attack of peritonitis; these were relieved by full doses of morphia. Soon after this the portion of omentum returned was found in the bottom of the wound, sloughing. It sloughed entirely away and the wound filled up by granulation. The patient made a good recovery, and in two weeks was considered out of danger. When the patient was convalescing there was a tendency to return of the hernia, and she had to wear a truss. At present writing the patient is perfectly well and enjoying excellent health.

---

CHLORAL, HYPODERMICALLY.—Dr. John Bartlett states, as a result of his experience, that as an hypodermic remedy, chloral is abominable. At the point injected there appears a black slough-like spot set in the skin, whitened, as if frozen. Intense inflammation of spot succeeds; no slough occurs.

